# An Over-dose of Homœopathy

**Published:** 1856-04

**Authors:** 

**Affiliations:** New York


*An Over-dose of Homœopathy.*

In one of our charitable institutions resided a lady of more than ordinary intelligence, not connected with the institution except as a boarder. She is a strenuous advocate of homœopathy. Some two years since, at considerable expense, she replenished her "homœopathic case." A favorite child in the establishment found her way into the room of this lady, and, as children are fond of doing, rummaged among the "baskets and boxes;" discovering some pretty little bottles, full of pretty little somethings, she began to draw the corks, child-like. Finding the little somethings nice and sweet, she continued eating them, until the lady, who was napping it on the bed, awoke, when, to her dismay, she discovered that the "little one" had transferred to her stomach ten bottles full of globules, and in her pocket she found the empty bottles. What was to be done? Only think! a little child's stomach filled with homœopathic fixings! The worthy matron of the institution, whose medical predilections belonged to quite another school, was immediately summoned, to whom was told the awful catastrophe. When asked what should be done, she coolly replied, "why, give her more, if she wants—they won't hurt her." But hours and days passed before the lady herself was satisfied that no harm was done; and glad enough was she to find that the little orphan had really lived through it. But did not such an over-dose of "medicine" vomit her? No. Nor purge her? No. Nor sweat her? No. Nor make her sleep? No. Nor make her sick in any way? No, all she wanted was more of the same sort. There was nux vomica, aconite, belladonna, cicuta, rhus, mercury, antimony, silex, oystershell, &c. The request of "let us say nothing about it," was strictly observed, and latterly had been scarcely thought of. The injunction would not probably have been raised, was it not for the following occurrence· Within a few weeks, several cases of scarlet fever have made their appearace in the institution, and two of them proved fatal. Death threatened a third one laboring under the congestive form of that fever. He was attended by a physician of the good old stamp. One of the governors, not Governor Clark, but a man of mighty mien—not a Know-nothing in his own estimation certainly, but a—homœopath, in his diurnal visit asked after the little sufferer, the lady boarder above referred to being present. He was informed that he was no better, and that the doctor thought he would die (he did not, however, die). The old gentleman preserved his usual dignity and calmness until this announcement, when both seemed to quit him at once, as he delivered himself somewhat after the following manner: "This won't do. This won't do. This crowding down medicines in such quantities is enough to kill the children without the scarlet fever. It must be stopped. It will not do," &c. &c. After he had fairly relieved himself, the worthy matron replied in substance, that she did not know what the gentleman meant by crowding down medicine; she had seen no such crowding as he alluded to—she was certain there was nothing of the kind here; the doctor was opposed to any such treatment. Indeed, the only case in which she had ever seen, in which she thought the child had been over-dosed, occurred some two years ago. She then related the case of the ten bottles, and appealed to the lady to confirm what she had stated, adding that the servants knew all about it at the time. Confusion dumfounded darkened two faces, while victory shed a glow of light over the other. Not another word was uttered. Not a question asked. The old gentleman's visit was cut abruptly short, and for several days it was not convenient for him to repeat it. MUM.

*New York, February* 21, 1856. [*Boston Med. and Surg. Journ.*

